# *In vitro* anticancer effects of frankincense and its nanoemulsions for enhanced cancer cell targeting

**DOI:** 10.3389/fphar.2025.1403780

**Published:** 2025-02-06

**Authors:** Rayya A. Al-Balushi, Aiswarya Chaudhuri, Raghuram Kandimalla, Ashanul Haque, Khalaf M. Alenezi, Mohd. Saeed, Mohammad Changez, Thuraya Al Harthy, Mohammed Al Hinaai, Samra Siddiqui, Ashish Kumar Agrawal, Farrukh Aqil

**Affiliations:** ^1^ Department of Basic and Applied Sciences, College of Applied and Health Sciences, A’Sharqiyah University, Ibra, Oman; ^2^ Department of Pharmaceutical Engineering and Technology, Indian Institute of Technology (BHU), Varanasi, India; ^3^ Brown Cancer Center, University of Louisville, Louisville, KY, United States; ^4^ Department of Chemistry, College of Science, University of Hail, Hail, Saudi Arabia; ^5^ Department of Biology, College of Science, University of Hail, Hail, Saudi Arabia; ^6^ College of Health Sciences, University of Buraimi, Al Buraimi, Oman; ^7^ Department Health Services Management, College of Public Health and Health Informatics, University of Hail, Hail, Saudi Arabia; ^8^ Department of Medicine, University of Louisville, Louisville, KY, United States

**Keywords:** frankincense oil, nanoemulsion, box-Behnken design, breast cancer, drug delivery

## Abstract

**Introduction:**

Frankincense has demonstrated promising *in vitro* anticancer activity. However, its conventional delivery methods face significant challenges due to limited oral bioavailability. To address these limitations, this study focuses on developing optimized nanoemulsions (NEs) of Frankincense oil (FO) to enhance its therapeutic efficacy.

**Methods:**

Frankincense resins were extracted and characterized using gas chromatography-mass spectrometry (GC-MS) and liquid chromatography-mass spectrometry (LC-MS), identifying key metabolites including isopinocarveol, α-thujene, p-cymene, carvone, germacrene A, and various methyl esters. FO-based nanoemulsions (FO-NEs) were prepared and optimized using a 3-factor, 3-level Box-Behnken Design (BBD), with 10% FO (v/v), 40% surfactant (cremophor EL), and co-surfactant (Transcutol P). The optimized FO-NEs were evaluated for particle size, polydispersity index (PDI), zeta potential, and morphology using scanning electron microscopy (SEM) and atomic force microscopy (AFM). Cytotoxicity, wound healing, mitochondrial membrane potential (MMP), and reactive oxygen species (ROS) assays were performed against breast cancer (MDA-MB-231, MDA-MB-231-TR) and lung cancer (A549, A549-TR, H1299) cell lines.

**Results:**

The optimized FO-NEs exhibited an average particle size of 65.1 ± 4.21 nm, a PDI of 0.258 ± 0.04, and a zeta potential of −22.3 ± 1.2 mV. SEM and AFM confirmed the spherical morphology of the FO-NEs. In vitro cytotoxicity studies revealed enhanced anticancer activity of FO-NEs (IC50 = 13.2 μg/mL) compared to free FO (IC50 = 22.5 μg/mL) against resistant breast cancer MDA-MB-231-TR cells. FO-NEs significantly improved cancer cell internalization, disrupted mitochondrial membrane potential, and increased ROS generation, leading to enhanced cytotoxic effects.

**Discussion:**

The results demonstrate that nanoemulsion-based delivery significantly enhances the bioactivity and cellular uptake of frankincense oil compared to its free form. FO-NEs exhibit potent anticancer activity, particularly against drug-resistant cancer cell lines, suggesting their potential as a viable strategy for improving the therapeutic efficacy of frankincense in cancer treatment.

## Introduction

Cancer continues to rank as the second leading cause of death worldwide, surpassed only by cardiovascular diseases, resulting in over 7 million fatalities annually (Siegel et al., 2022). Among various cancer types, breast cancer is the most commonly diagnosed cancer in women, contributing approximately 25.8% of new cases in the year 2020, and is considered the second–leading cause of cancer–related deaths (Giaquinto et al., 2022). In the United States, it is estimated that 313,510 women will be diagnosed with breast cancer and 42,780 will die from the disease in 2024 (Siegel et al., 2024). Chemotherapy remains a cornerstone of breast cancer treatment, with standard regimens including aromatase inhibitors (e.g., anastrozole, letrozole) and CDK 4/6 inhibitors for advanced hormone receptor-positive cases (Rossi et al., 2019). Other drugs like paclitaxel, docetaxel, carboplatin, capecitabine, gemcitabine, cisplatin, vinorelbine, etc., are often used in combinations (Waks and Winer, 2019). However, these treatments are often accompanied by moderate to severe side effects, including organ failure, alopecia, diarrhea, vomiting, and bone marrow cell depletion, which in turn causes exhaustion (because of low red blood cells), bleeding (related to low platelet count), and infections (due to low white blood count) and other secondary diseases (Sharma et al., 2010). Drug resistance further complicates treatment, driving the need for novel therapeutic approaches. In response to the above-mentioned challenges, there is an increasing interest in the discovery of new synthetic, semi-synthetic and natural products (NPs) based anticancer agents. NPs have been a substantial source of drugs for centuries. Today, a wide array of plant-based compounds and their semi-synthetic derivatives have been identified and are currently being utilized clinically (Asma et al., 2022).

Among the diverse and extensive collection of plant species, the frankincense plant (*Boswellia sacra* Flueck), a perennial plant, has garnered a significant interest from the researchers. Not only is it used in traditional medicinal, but it also substantially influences the economy (Miran et al., 2022; Khalifa et al., 2023). Various works demonstrating that the gum or frankincense or olibanum (*abbreviated as FO from here onwards*), a secretion from the bark of *B. sacra*, is a valuable herbal source of pharmaceuticals is available in literature. It has been documented to exhibit antiproliferative properties against various types of cancer (Akl and Sylvester, 2013; Hakkim et al., 2019; Abd-Rabou and Edris, 2022; Farahani et al., 2023). Chemically, FO is comprised of terpene, sesquiterpene, and diterpene.

*Boswellia sacra* Flueck. has demonstrated promising anticancer properties, particularly against breast cancer. Research indicates that extracts from this plant can inhibit the proliferation of breast cancer cells. A recent study reported that *Boswellia* extract significantly reduced tumor proliferation in breast cancer patients, suggesting its potential as an adjunctive therapy (Valente et al., 2024). Additionally, *Boswellia sacra* extracts have been found to enhance the effects of conventional chemotherapeutic agents. Boswellic acids, active constituents of this plant, have been shown to potentiate the anticancer effects of other drugs, suggesting a synergistic potential in cancer treatment regimens (Liu and Duan, 2009).

Boswellic acids also modulate various molecular pathways involved in cancer progression. These compounds can inhibit enzymes like 5-lipoxygenase, which are implicated in tumor growth and metastasis (Trivedi et al., 2023). These bioactive triterpenoids suppress tumor growth by inhibiting key signaling pathways such as NF-κB, STAT3, and PI3K/AKT, which are critical for cancer cell survival and proliferation. Additionally, they induce apoptosis by modulating pro-apoptotic and anti-apoptotic proteins such as Bax and Bcl-2. Boswellic acids also demonstrate anti-angiogenic and anti-metastatic properties, further limiting tumor progression. These metabolites exhibit significant potential in reducing the growth of various cancers, including breast and colon cancer, while exhibiting minimal toxicity to normal cells (Trivedi et al., 2023). Despite its therapeutic potential, *B. sacra* Flueck clinical applications are limited by poor solubility, bioavailability, and potency.

To overcome problem of low bioavailability, toxicity and increase the potency of a drug candidate, nanotechnology has emerged as a promising tool (Sahu et al., 2021). Various nanoformulations of doxorubicin (DaunoXome^®^ and Doxil^®^) and paclitaxel (Abraxane^®^) are clinically used for breast cancer chemotherapy (Sharma et al., 2010). Among different types of nano-systems, nanoemulsions are composed of a mixture of oil and water that is thermodynamically or kinetically stabilized via an emulsifier or surfactant. They usually range less than 200 nm in size and are reported to have the ability to reduce drug resistance and increase targetability (Vaz et al., 2020). Various researchers have demonstrated that the nanoemulsions incorporating NPs extract(s) show improved biological profile due to improved bioavailability and targetability of the extract/compound (Ranjbar et al., 2023). Recent research suggests that frankincense nanoformulation enhances antileishmanial and anticancer activities (Babaei et al., 2024; Seku et al., 2024).

Considering the importance of frankincense in cancer research and limited bioavailability, we report herein the preparation of a nanoemulsion of frankincense oil (FO) and its cytotoxic efficacy against breast cancer (MDA-MB-231 and MDA-MB-231-TR) and lung cancer (A549, A549-TR, and H1299) cell lines. We perform a series of assays, including cytotoxicity, wound healing, mitochondrial membrane potential (MMP), and reactive oxygen species (ROS) assays, to assess the improved therapeutic potential of the FO nanoemulsion compared to conventional FO.

## Methods and materials

### Materials and instrumentation

All solvents used for isolation and purification were of ACS reagent grade (Sigma-Aldrich Chemical Co., Germany). Frankincense resin was obtained commercially from the local market (Ibra, Sultanate of Oman). The origin of samples was from Oman (*Omani luban*) and was authenticated by local and the authors, and a voucher specimen has been deposited. Tween 80, and Tween 20 were purchased from Merck, India. Poloxamer 188 was obtained from BASF Ltd. (Navi Mumbai, Maharashtra, India). PEG 6-Caprylic/Capric glycerides and PEG-8-Caprylic/Capric glycerides were obtained from Parchem Fine and Specialty Chemicals, NY, United States. Cremophor EL was obtained from Biomall, and transcutol P was obtained as a gift sample from Gattefossé. 3-(4,5-dimethylthiazol-2-yl) −2,5-diphenyltetrazolium bromide (MTT), and fetal bovine serum (FBS) were purchased from Thermo Scientific, Waltham, MA, United States. Dulbecco’s Modified Eagle Medium (DMEM), antibiotic, anti-mitotic, and trypsin solutions were purchased from Hi Media, Mumbai, India. Methanol, acetonitrile, and tween 80 were purchased from Merck, India. Coumarin-6 was purchased from Sigma Aldrich, St Loius, MO, United States, and H_2_DCFDA was obtained from Hi Media, Mumbai, India. All the chemicals used are of analytical grade. The alterations within the functional groups of the samples were analyzed using FT-IR spectroscopy (Nicolet iS5, THERMO Electron Scientific Instruments LLC). The FTIR spectrum of excipients and the FO-NEs were obtained by attenuated total reflectance (ATR). The FTIR spectra were scanned over the range of 4,000–400 per cm^−1^ for 32 scans per cycle (Venkateshwarlu et al., 2010; Farshi et al., 2019). Chromatographic analyses were carried out using performed gas chromatography (GC-MS) and liquid chromatography–mass spectrometry (LC-MS) techniques. GC-MS data was collected on an Agilent (United States) equipped with a 5% phenyl methyl silox (5MS) capillary column (dimension: 30 m × 250 μm × 0.25 μm). The column oven temperature was initially set at 50°C and increased by 1°C/min to 120°C with a hold of 5 min, then increased to 250 with 2°C/min. The injector and MS transfer line temperatures were kept at 250°C. Helium was used as a carrier gas at a constant 1 mL/min flow rate. Mass spectra were acquired and processed by Agilent MassHunter workstation and Agilent MassHunter qualitative analysis software. The metabolites were identified by comparing their retention times and mass spectra available in spectral databases. LC-MS (Q-TOF) was carried out on Agilent 6,530 with C_18_ column (dimensions: 4.6 mm × 150 mm × 5 μm). Pre-concentrated samples (15.00 µL) were injected using Hamilton syringes into the injector. The oven temperature was 35°C, and the gradient mobile phase consisted of 30% A:70% B (where A = 100% H_2_O+ 0.1% formic acid +10 mM ammonium formate and B = 100% acetonitrile +0.1% formic acid +10 mM ammonium formate). The flow rate was set 0.5 mL/min. The software program Agilent Masshunter was used belongs to Agilent Technologies.

## Methods

### Frankincense resin extraction

Omani frankincense (*B. sacra* Flueck) was extracted by shaking and mixing 25 g of resin in n-hexane (C_6_H_14_) overnight at room temperature, followed by filtration. It was repeated thrice, and the combined extracts were concentrated using rotatory evaporation at room temperature. The resulting viscous oily liquid (frankincense crude oil or the hexane extract) was stored at −20°C until further analysis.

### Screening of surfactants and co-surfactants

Surfactants and co-surfactants were screened for their emulsifying ability. For screening of surfactant, 0.5 g of each surfactant (Tween 80, Tween 20, Cremophor EL, Poloxamer 188, PEG 6-Caprylic/Capric glycerides, and PEG-8-Caprylic/Capric glycerides) were blended with 0.5 g FO. Each mixture was heated in a water bath at 37°C for uniform homogenization. Each mixture (0.2 g) was then reconstituted with 20 mL of deionized water and allowed to stand for 1 h. A clear or tint of blue appearance was observed for the formation of nanoemulsion by reporting the number of flask inversions, followed by the analysis of % transmittance at 638.2 nm against distilled water. Similarly, for the screening of co-surfactant, 0.25 g of each co-surfactant (PEG 400, PEG 600, Transcutol P, Propylene glycol, and Span 80) were blended with 0.25 g selected surfactant and 0.5 g FO. The mixtures were then heated in a water bath at 37°C for uniform homogenization, followed by reconstituting 0.2 g of the mixture with 20 mL of deionized water. The number of flask inversions was noted, followed by the analysis of per cent transmittance at 638.2 nm against distilled water. The surfactant and co-surfactant exhibiting a minimum number of flask inversions with maximum per cent transmittance were chosen to prepare nanoemulsion (Date and Nagarsenker, 2008).

### Ternary phase diagram

A ternary phase diagram was constructed to determine the ratios of oil:Smix (surfactant and co-surfactant) for preparing optimized nanoemulsion. The Smix were mixed in different weight ratios (1:1, 2:1, 3:1, 4:1, and 1:2). Further, oil and Smix were mixed uniformly at specific ratios in nine proportions, namely 1:9, 2:8, 3:7, 4:6, 5:5, 6:4, 7:3, 8:2, and 9:1. Then, a small amount of water in 0.5% (by weight) increment was added into the oil:Smix mixture under mild agitation and allowed to equilibrate. The titration was stopped, and the water volume was recorded when the nanoemulsion appeared transparent. The ratio showing increased nanoemulsion region was selected to prepare nanoemulsion (Nasr et al., 2016).

### Nanoemulsion preparation and optimization

Nanoemulsion (NEs) was prepared using a high-energy method, which includes high-speed homogenization followed by ultrasonication (Venkateshwarlu et al., 2010) with slight modification. Briefly, the required amount of Smix was added in the aqueous phase, and it was allowed to stir. After complete solubilization of Smix, it was subjected to a high-speed homogenizer (IKA T25 digital, Ultra Turrax), and the required amount of FO was added dropwise and allowed to homogenize for 15 min, at 15,000 rpm under ambient temperature, followed by sonication via probe sonicator for 5 min. The prepared nanoemulsion was then allowed to cool at room temperature and stored at 4°C until further characterization.

Optimization of Frankincense oil (FO) was performed using Design-Expert^®^ software v. 9.0.1 trial (Stat-Ease Inc., Minneapolis, MN, United States), where 3-factor, 3-level Box Behnken Design (BBD), a type of response surface methodology (RSM) experimental design was employed for the assessment. The formulation (FO and Smix concentration), and condition (sonication amplitude) that give the optimum nanoemulsion were determined using the RSM technique (Supplementary Table ST1) where the independent parameters include A: concentration of oil (5–15%v/v), B: concentration of Smix (20%–40% v/v), and C: sonication amplitude (20%–70%), and dependent parameters or response includes Y_1_: particle size (nm), and Y_2_: polydispersity index (PDI). Seventeen experimental runs were generated (Supplementary Table ST2). A quadratic equation was used to fit the experimental results into the response surface regression procedure.

### Determination of particle size, PDI, and zeta potential

The particle size, polydispersity index (PDI), and zeta potential of the FO-NEs were measured by the dynamic light scattering (DLS) method. In prior experiments, the samples were diluted 100 times with distilled water and measured for particle size, PDI, and zeta potential using Zeta – sizer (DelsaTMNano, Beckman coulter, Brea, CA, United States). Each FO-NE sample was measured in triplicates (Rehman et al., 2020).

### Surface morphology

Surface morphology, roughness, topography, and height profile of the FO-NEs were characterized using scanning electron microscopy (SEM), atomic force microscopy (AFM), and transmission electron microscopy (TEM). For SEM, FO-NEs (∼30 μL) were diluted 600 times with distilled water, which was then placed onto the clean glass slides and kept aside for drying. After drying, the glass slides were coated with gold and observed in high-resolution SEM 175 (Nova Nao SEM 450, FEI, Hillsboro, OR, United States) at 15.0 kV (Farshi et al., 2019). For AFM, 10 μL FO-NEs were diluted with distilled water in a 1:1,000 v/v ratio, which was then deposited onto a glass slide and allowed to dry for the removal of excess water. The glass slide was then subjected to an atomic force microscope (NTEGRA Prima, NT-MDT Service and Logistics Ltd.). The nominal resonant frequency of the cantilever and the nominal force constant used is 150 kHz, and 5.1 N/m, respectively. The Image Analysis 2.2.0 (NT-MDT) software was employed for processing the AFM data (Dokovic et al., 2021). For TEM, the FO-NEs were first diluted and then a drop of the diluted dispersion of the nanoemulsion was dropped onto the carbon-coated grid, air-dried, and observed under a transmission electron microscope (Tecnai G2 20 TWIN, FEI Company of United States (S.E.A) PTE, LTD.), operated at 120 kV (El-Mancy et al., 2021).

### Thermodynamic stability studies

The optimized FO-NEs were subjected to thermodynamic stability studies comprised of centrifugation, heating cooling cycle, and freeze-thaw cycle. In the centrifugation test, the optimized FO-NEs were subjected to 3,000 rpm for 30 min. In the heating and cooling test, the optimized FO-NEs were subjected to six cycles of heating and cooling at 4°C–40°C for 48 h. In freeze-thaw study, the optimized formulation was subjected to a temperature between −20°C and 25°C for 48 h. The optimized FO-NEs were analyzed for instability such as phase separation, creaming, and cracking (El-Mancy et al., 2021).

### Cell culture

Breast cancer (MDA-MB-231) and lung cancer (A549 and H1299) cells were obtained from the American Type Culture Collection (ATCC, Manassas, VA). MDA-MB-231 cells were cultured in L-15 medium, while A549 and H1299 cells were cultured in RPMI and DMEM media, respectively. All media were supplemented with 10% fetal bovine serum and 1% antibiotics (penicillin/streptomycin). Breast cancer cells were incubated at 37°C without CO_2_ supply, whereas lung cancer cells were incubated at 37°C in a humidified incubator with 5% CO_2_. The drug resistance MDA-MB-231-TR and A549-TR cells were grown under the same culture conditions as their sensitive counterparts, MDA-MB-231 and A549.

### *In–vitro* cytotoxicity assay (MTT assay)

The antiproliferative activity of the FO and its nanoemulsion against various cancer cells was assessed by MTT assay, as described elsewhere (Kausar et al., 2012; Ogunc et al., 2017). Briefly, breast cancer (MDA-MB-231 and MDA-MB-231-TR) and lung cancer (A549, A549-TR, and H1299) cells were seeded in a 96-well plate and treated with various concentrations of FO and FO-NEs for 24–72 h. At the end of treatment, the media was replaced with media containing MTT (0.5 mg/mL) and incubated for 2 hours, followed by solubilization of formazan crystals with DMSO and spectrophotometric measurement at 570 nm. IC_50_ values were calculated using Calcusyn software Version 2.0 (Biosoft, United Kingdom).

### Wound healing assay

MDA-MB-231 cells (10^4^ cells/well) were seeded in the culture-insert two well (Ibidi, Lochhamer Schlag, Gräfelfing Germany). The cells were then incubated for 24 h. After incubation, a wound was created by removing the culture inserts with sterile forceps. The cells were then washed with PBS three times, followed by the incorporation of fresh media consisting of FO and FO-NEs, and incubated for up to 24 h. The wounds were visualized at 0, 12, and 24 h, and microphotographs were taken at each time point. Further, the wounded regions for each photograph were determined quantitatively using ImageJ software (Kyakulaga et al., 2018).

### Mitochondrial membrane potential (MMP) assay

The alterations within the mitochondrial membrane potential (MMP) due to FO were determined by JC-1 dye (Invitrogen, Thermo Scientific, United States). Briefly, 1 × 10^5^ MDA-MB-231 cells/well were seeded in 12 well plates and incubated for 24 h. After 24 h, the media was aspirated and fresh media was added consisting of FO and FO-NEs and incubated for 24 h. After the completion of the treatment, the cells were washed with PBS and treated with 5 μg/mL JC-1 dye in culture media, followed by incubation for 20 min. After 20 min, the cells were again washed three times with PBS to remove the unbound JC-1 dye. The images of the treated cells were analyzed using a fluorescence microscope (Olympus BX53, Tokyo, Japan) (Shanmugapriya et al., 2019).

### Analysis of reactive oxygen species (ROS) production

The production of intracellular ROS was assessed using DCFDA dye. Briefly, MDA-MB-231 cells (1 × 10^5^ cells/well) were seeded in 12 well plates and incubated overnight. After 24 h, media was aspirated, and fresh was incorporated into the wells consisting of FO and FO-NEs and incubated for 48 h. The cells were then permeabilized using methanol for 1 h, followed by the addition of DAPI (10 μg/mL) and DCFDA (10 μg/mL) and incubated for 30 min. The solution was removed, and cells were washed three times with PBS. The fluorescence intensity was observed under the fluorescence microscope (Olympus BX53, Tokyo, Japan). For positive control, the cells were treated with 0.05% of H_2_O_2_, for 2 h before the addition of methanol (Shanmugapriya et al., 2019).

### Statistical analysis

All the data were measured as mean ± SD. Statistical analysis was performed using GraphPad Prism 5.01 software. The significance level was analyzed employing analysis of variance (ANOVA), where p-value <0.05 was considered statistically significant.

## Results and discussions

### Extraction and chemical characterization of FO extract

**CHART 1:**
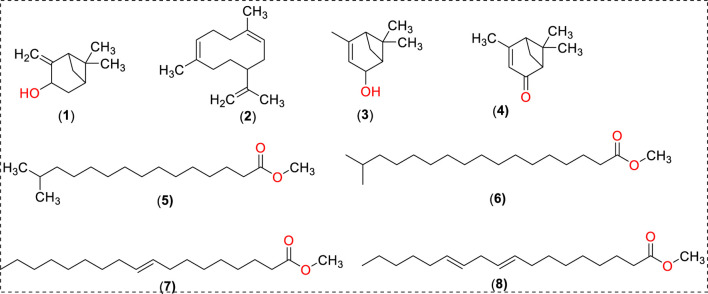
Putative identification of phytochemicals identified by GC-MS and LC-MS.

The active constituents found in *Boswellia spp*. have been extensively studied (Camarda et al., 2007; Al-Yasiry and Kiczorowska, 2016; DeCarlo et al., 2019; Alotaibi et al., 2021; DeCarlo et al., 2022). A typical composition of *B. sacra* Flueck plant extract includes mono-, sesqui-, and diterpenes and their oxygenated derivatives. Various methods and solvent systems have been reported to extract these phytochemicals. Indeed, hydro-distillation has been the method of choice as it can extract polar (mainly) phytochemicals from the resin. Some researchers also reported that hydro distillate extracts contain constituents that possess biological activities like α-pinene, α-thujene, limonene, (E)-b ocimene, and octyl acetate (Mertens et al., 2009). In the present study, we adopted a cold solvent extraction method using hexane as a solvent to extract nonpolar and volatile metabolites from the frankincense resin and study their bioactive nanoformulation. GC-MS profile of FO extract showed peaks between 22.16 and 93.32 min tentatively assigned to isopinocarveol, 1,5-dimethyl-8-(prop-1-en-2-yl)cyclodeca-1,5-diene, 4,6,6-trimethylbicyclo [3.1.1]hept-3-en-2-ol, 4,6,6-trimethylbicyclo [3.1.1] hept-3-en-2-one, methyl 14-methylpenta decanoate, methyl 16-methylheptadecanoate, methyl octadec-9-enoate, methyl-octadeca-9,12-dienoate, α-thujene, *p*-cymene, carvone, α-cubebene, germacrene A, and others (Chart 1) (Table 1; Supplementary Figure S1). The presence of phytochemicals as mentioned above is also supported by the FTIR-ATR spectrum of the crude extract (Supplementary Figure S2). The spectrum shows a vibrations band attributed to OH, Csp^3^-H stretching (alkyl), C=O stretching (ester), and C=C stretching (alkene), as reported by others (Azzazy et al., 2022). In addition to the GC-MS, attempts have also been made to analyze the phytochemicals present in the extract by LC-MS (positive mode) using a gradient mobile phase (Chart 1). LC-MC analysis of the crude extract revealed a trace of α/β-boswellic acids, 3-*O*-acteoxy treicalic acid isomers, 3-O-acetyl-oleanolic acid, fragments of 11-hydroxyboswellic acid and 3-hydroxytirucellic acid. (Supplementary Figure S3) (Mannino et al., 2016). It should be noted that the possibility that several other metabolites evaded detection, co-elution or misidentification cannot be ruled out and more sophisticated techniques would be required to get the exact composition of the FO extract.

**TABLE 1:** GC-MS analysis of the FEOs, elucidation of empirical formulas and putative identification of each metabolites.

S. No.	Identification	Mol. Form	M.W. (Da)	RT (min.)	Peak area (%)
1	Isopinocarveol	C_10_H_16_O	152.24	21.54	1.02
2	1,5-dimethyl-8-(prop-1-en-2-yl)cyclodeca-1,5-diene	C_15_H_24_	204.36	22.16	14.84
3	4,6,6-Trimethylbicyclo [3.1.1]hept-3-en-2-ol	C_10_H_16_O	152.24	26.56	1.3
4	4,6,6-trimethylbicyclo [3.1.1]hept-3-en-2-one	C_10_H_14_O	150.22	30.97	5.94
5	Methyl 14-methylpentadecanoate	C_17_H_34_O_2_	270.46	46.03	4.06
6	Methyl 16-methylheptadecanoate	C_19_H_38_O_2_	198.29	50.01	35.18
7	Methyl-octadec-9-enoate	C_19_H_36_O_2_	296.27	53.05	1.29
8	Methyl-octadeca-9,12-dienoate	C_19_H_34_O_2_	294.18	55.12	1.73

### Screening of surfactant and co-surfactant

All the surfactants employed in the study were non-ionic and hydrophilic, suggesting the surfactants are safe and biocompatible. Moreover, it was observed that the hydrophilic surfactants with HLB values >10 are considered superior in forming fine and uniform emulsion globules with large surface areas, thereby facilitating the rapid release of drugs and their absorption into the systemic circulation (Nemichand and Laxman, 2017). Surfactants are pivotal in developing nanoemulsions, as they can emulsify oil and form a monophasic system. Depending upon the emulsification ability, the surfactants were screened based on percent transmittance and the number of flask inversions. Table 2 indicates the % transmittance (%T) and the number of flask inversions of various surfactants employed to prepare the dispersion. It was observed that FO exhibited the highest % transmittance with cremophor EL (69.85 ± 1.29), and the least number of flask inversions (i.e., 7), indicating its superior capacity in emulsifying the FO into the aqueous phase (Table 2). Also, it was inferred that the increased HLB value of cremophor (12–14) corresponds to its hydrophilicity, which aids in decreasing the interfacial tension and entropy and creating a rapid dispersion of oil globules within the aqueous phase, thereby facilitating a microscopic o/w nanoemulsion.

**TABLE 2:** Transmittance (%) and number of flask inversion of different surfactants.

Surfactants	% Transmittance^a^	No. flask inversion
Tween 80	14.60 ± 0.64	30
Tween 20	26.00 ± 1.25	16
Cremophor EL	69.85 ± 1.29	7
PEG 6-Caprylic/Capric glycerides	25.00 ± 0.68	12
PEG 8-Caprylic/Capric glycerides	17.52 ± 0.72	24

^a^ Values are expressed as mean ± S.D., n = 3.

Similarly, different co-surfactants were selected on the same parameters as the surfactants. It was demonstrated that the co-surfactants are incorporated to improve the emulsification ability as they increase the spontaneity of forming the nanoemulsion. Also, the addition of co-surfactants along with surfactants helps in occupying the voids left by the long chain surfactants at the interfacial surface and, as a result, decreases the bending stress within the oil-water interface, causing the formation of a uniform monophasic system. Table 3 collects the transmittance (%T) and the number of flask inversions of various co-surfactants employed to prepare the dispersion. It was observed that Transcutol P showed a clear mixture with FO, and cremophor EL, with the highest % transmittance (89.05 ± 0.35), and the lowest number of flask inversions (i.e. 10).

**TABLE 3:** % Transmittance and the number of flask inversion of different co-surfactants.

Co-surfactants	% Transmittance*	No. of flask inversion
PEG 400	67.78 ± 2.17	34
PEG 600	67.70 ± 1.06	30
Transcutol P	89.05 ± 0.35	10
Propylene glycol	66.81 ± 0.57	24
Span 80	19.01 ± 0.38	45

* % Transmittance is expressed as mean ± S.D., n = 3.

**FIGURE 1:**
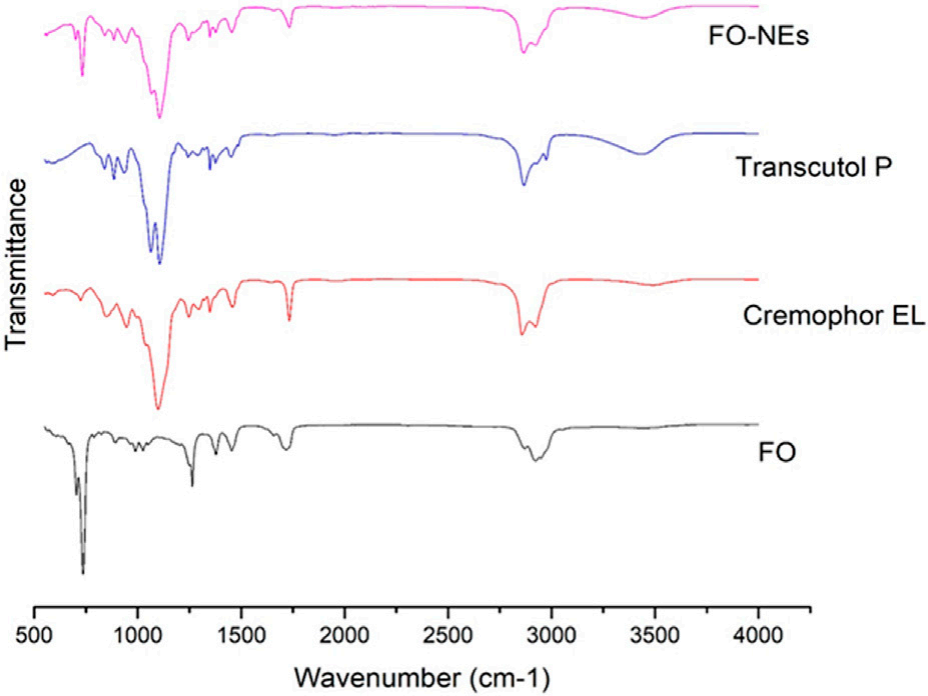
Fourier-transform infrared spectroscopy (FT-IR) of FO, and FO-NEs. The figure demonstrates that all three of the distinctive peaks for FO, transcutol P, and cremophor EL are present in FO-NEs.

FTIR spectra were obtained to identify the functional group and to evaluate the compatibility between the metabolites of the NEs, in the form of the appearance of new peaks or the disappearance of existing peaks. As shown in Figure 1, FO-NEs exhibit all the characteristic peaks of FO, transcutol P, and cremophor EL indicating no incompatibility between the metabolites of the NEs.

### Ternary phase diagram

**FIGURE 2:**
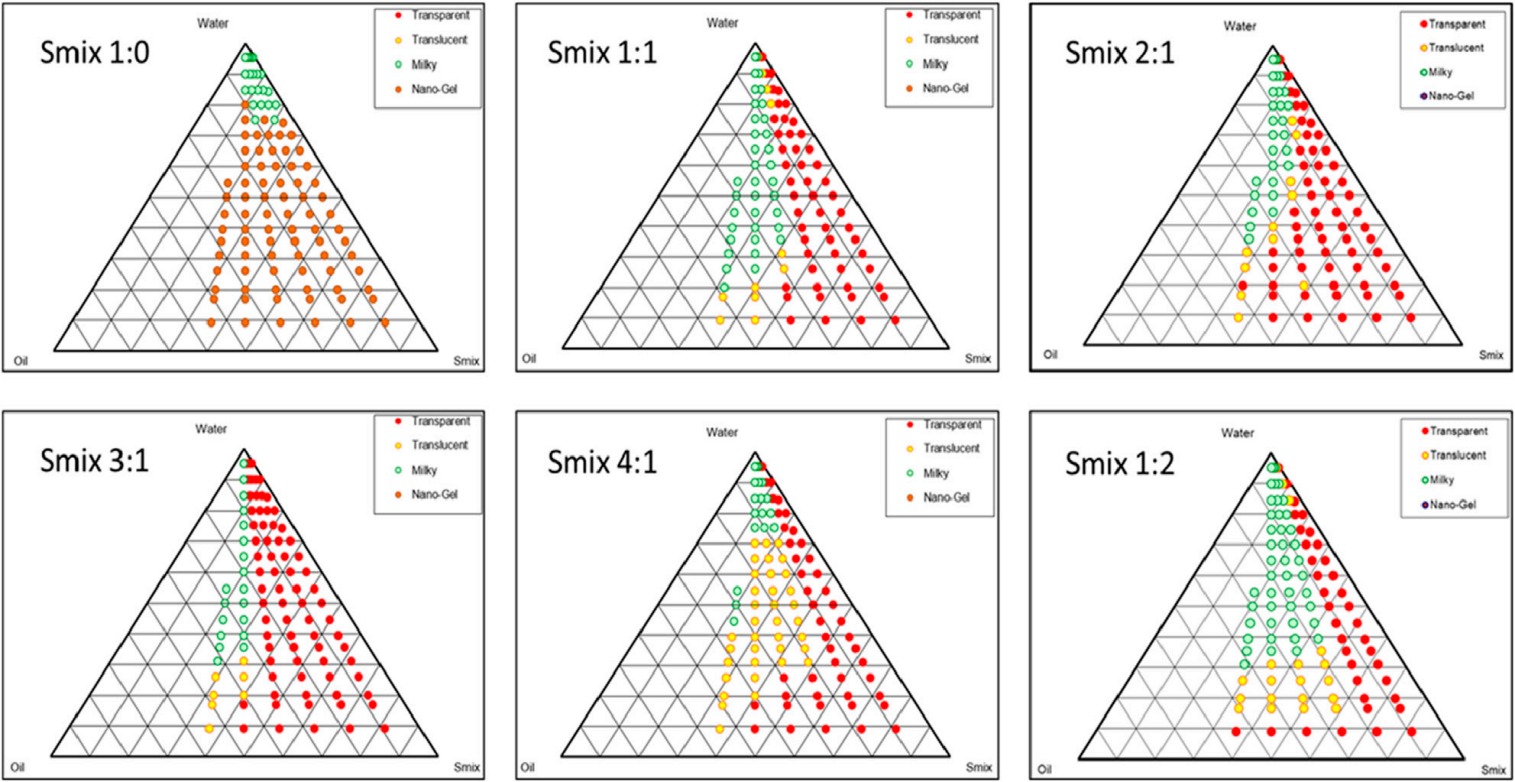
Ternary phase diagrams at Smix ratio: 1:0, 1:1, 2:1, 3:1, 4:1, and 1:2. Red dots indicates transparent region, yellow dots indicates translucent region, green dots indicates milky region and pink dots indicates nano-gel region.

Ternary phase diagrams were constructed to recognize the nano-emulsifying regions and to identify optimum concentrations of oil (FO), surfactant, and cosurfactant for the formulation of NEs. Figure 2 shows the ternary phase diagram at different Smix ratios (Anwer et al., 2014). It was demonstrated that the greater the nanoemulsion area, the superior the nano-emulsifying efficiency of the Smix. In other words, it can be implied that the existence of the nanoemulsion area depends on the capacity of the ratio of Smix, that is required to solubilize the oil phase. The ternary phase diagram revealed that the *Smix* ratio of 3:1 exhibited a larger nanoemulsion area compared to other Smix ratios.

### Nanoemulsion preparation and optimization

BBD was employed to analyze the identified parameters and optimize the FO-NEs statistically. Also, applying such experimental designs avoids wasting time in formulating a large number of formulations aimlessly. From the BBD, we obtained 17 experimental runs (formulations) carried out for the optimization, and all the results are described in Supplementary Table ST2. The 17 formulations prepared for optimization were subjected to DLS for particle size and PDI, from where we obtained the particle size (Y_1_), and PDI (Y_2_) between 20 and 150 nm, and 0.109 to 0.386, respectively. The predicted responses were depicted by the quadratic equation using the coded variables:

$$Y_{1}=60.20+16.50A\;–\;25.25B\;–\;31.75C\;–\;8.00\text{AB }–\;4.50\text{AC}+23.00\text{ BC}+10.15\;A^{2}+4.65B^{2}+9.15C^{2}$$

$$Y_{2}=0.2454+0.0061A\;–\;0.0529B\;–\;0.0380C\;–\;0.0110\text{AB }–\;0.0253\text{AC}+0.0912\text{BC}+0.0087A^{2}+0.0152B^{2}+0.0179C^{2}$$

The appropriateness of the BBD was indicated by the high values of the regression coefficient (Table 4), which are obtained for all the responses (R^2^ = 0.9329 and 0.9124 for Y_1_, and Y_2,_ respectively). Furthermore, from the lack-of-fit data (Table 5), it was observed that there exists no evidence of the inadequacy of the mathematical model employed and was found to be significant (p < 0.05). P-value <0.05 and a regression coefficient close to 1 demonstrate a more significant effect on response variables for any of the terms in the model.

**TABLE 4:** Probability values (p-values), error lack of fit, and regression for each response.

Response	R^2^	Lack of fit	p-values								
			Linear			Quadratic			Interaction		
			A	B	C	A^2^	B^2^	C^2^	AB	AC	BC
Y_1_	0.9329	0.3380	0.0121^a^	0.0013^a^	0.0003^a^	0.1773	0.5141	0.2184	0.2871	0.5376	0.0129^a^
Y_2_	0.9124	0.1806	0.6023	0.0022^a^	0.0117^a^	0.5915	0.3587	0.2841	0.5107	0.1557	0.0007^a^

^a^ Significant p < 0.05. R2: coefficient of determination; A: oil phase concentration (% v/v); B: smix concentration; C: sonication amplitude (%); Y_1_: droplet size (nm); Y_2_: PDI.

**TABLE 5:** Predicted and experimental values obtained from the response optimization of BBD.

Responses	Predicted value	Experimental value^a^
Particle size (nm)	60.2	65.1 ± 4.21
PDI	0.2454	0.258 ± 0.04

^a^ Mean ± SD of five determinations; SD, standard deviation.

**FIGURE 3:**
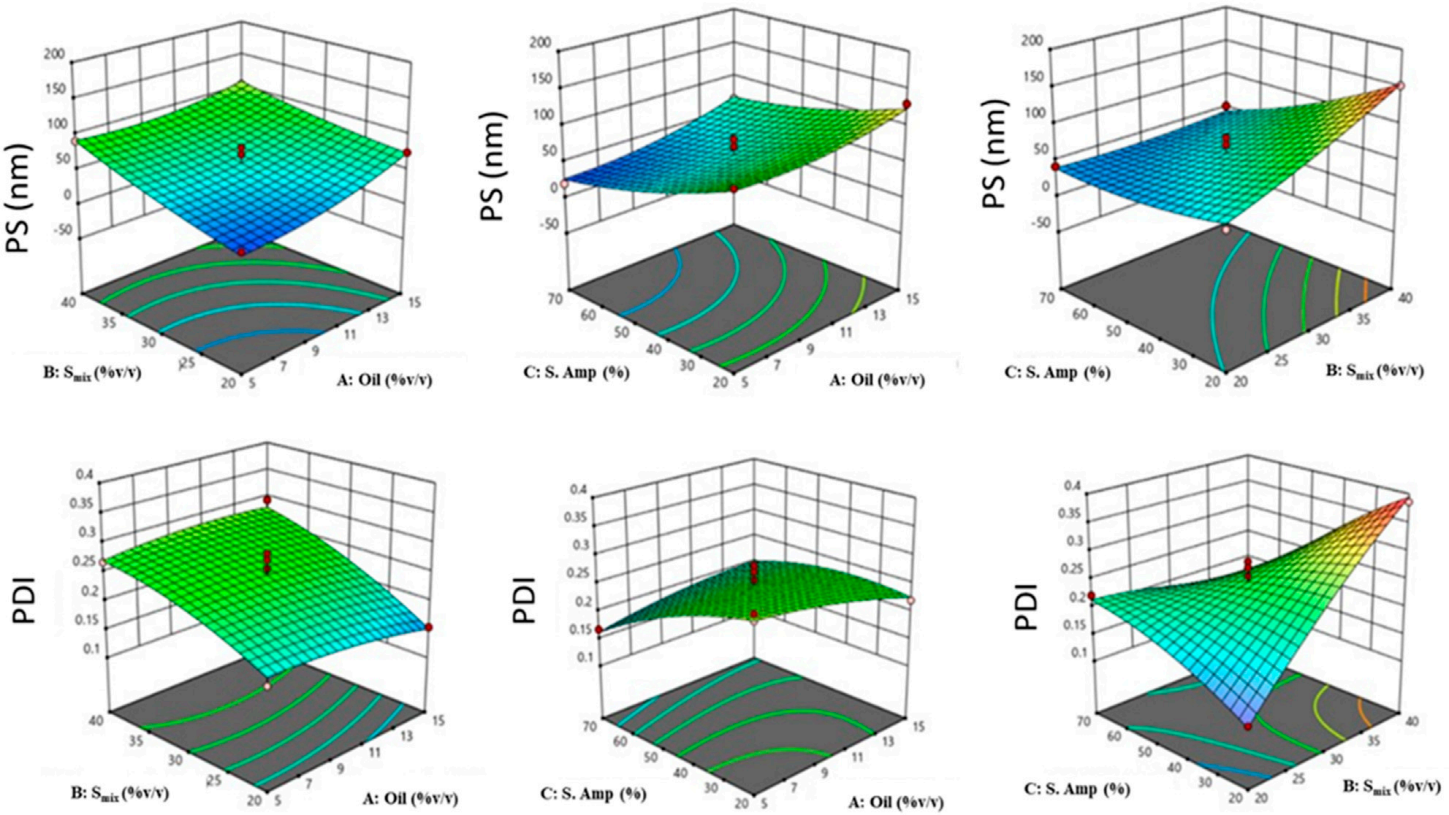
3D-response surface plots depicting the effect of factors: oil concentration (%v/v), Smix concentration (%v/v), and sonication amplitude (%) on responses: PS (Y1, nm) [Top], and PDI (Y2) [Bottom].

Further, response surface plots were plotted for each response condition (Figure 3), and composite desirability functions were also assessed to optimize the nanoemulsion. The optimized conditions were 10% oil phase (v/v), 40% Smix (v/v), and 45% sonication amplitude with a 0.9453 calculated composite desirability index. Further, the optimum formulation was developed in five replications to validate the predictive ability of the mathematical model’s equation. Table 5 shows the predicted and experimental values of the five replications and reveals an excellent predictive capacity of the model.

### Particle size, PDI, and zeta potential

**FIGURE 4:**
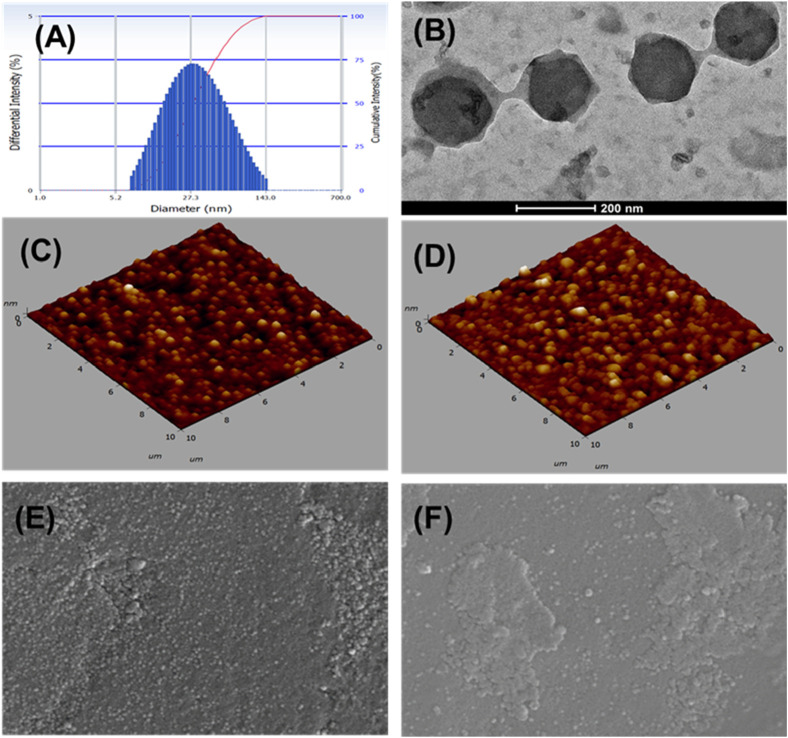
Characterization of FO-NEs. Particle size distribution **(A)**, and surface morphology via TEM **(B)**, AFM **(C, D)**, and SEM **(E, F)**.

The optimized FO-NEs showed an average particle size and PDI of 65.1 ± 4.21 nm and 0.258 ± 0.04, respectively (Figure 4A). Zeta potential was measured to analyze the surface charge on the NEs, which further indicates the stability of the NEs. The optimized FO-NEs had a zeta potential of – 22.3 ± 1.2 mV. It was further depicted that the stability of NEs corresponds to the magnitude of charge present on the surface of the NEs, which ranges from −30 mV to 30 mV to form stable NEs. Moreover, it was observed that more significant negative or positive zeta potential causes the NE globules to provide a repulsive force towards each other, creating a stable colloidal dispersion.

### Surface morphology

The surface morphology of FO-NEs was found to be spherical, as confirmed by transmission electron microscopy (Figure 4B), atomic force microscopy (AFM) (Figures 4C, D) and scanning electron microscopy (SEM) (Figures 4E, F), with slight variation in size compared to the size obtained from the particle size analyzer.

### Thermodynamic stability studies

The optimized FO-NEs demonstrated notable thermodynamic stability across all tested conditions. In the centrifugation test at 3,000 rpm for 30 min, the formulation showed no signs of phase separation or sedimentation, indicating strong mechanical stability. The heating and cooling cycles, which ranged from 4°C to 40°C over 48 h, also revealed no instability, as the FO-NEs maintained their integrity without phase separation, creaming, or cracking. Similarly, the freeze-thaw cycles between −20°C and 25°C for 48 h did not induce any destabilization. These results underline the formulation’s ability to resist various stress conditions, making it suitable for practical applications where stability under temperature fluctuations and mechanical stress is crucial. The findings confirm that the FO-NEs are well-formulated for both short-term and long-term storage.

## *In vitro* studies

### Cytotoxicity study

**FIGURE 5:**
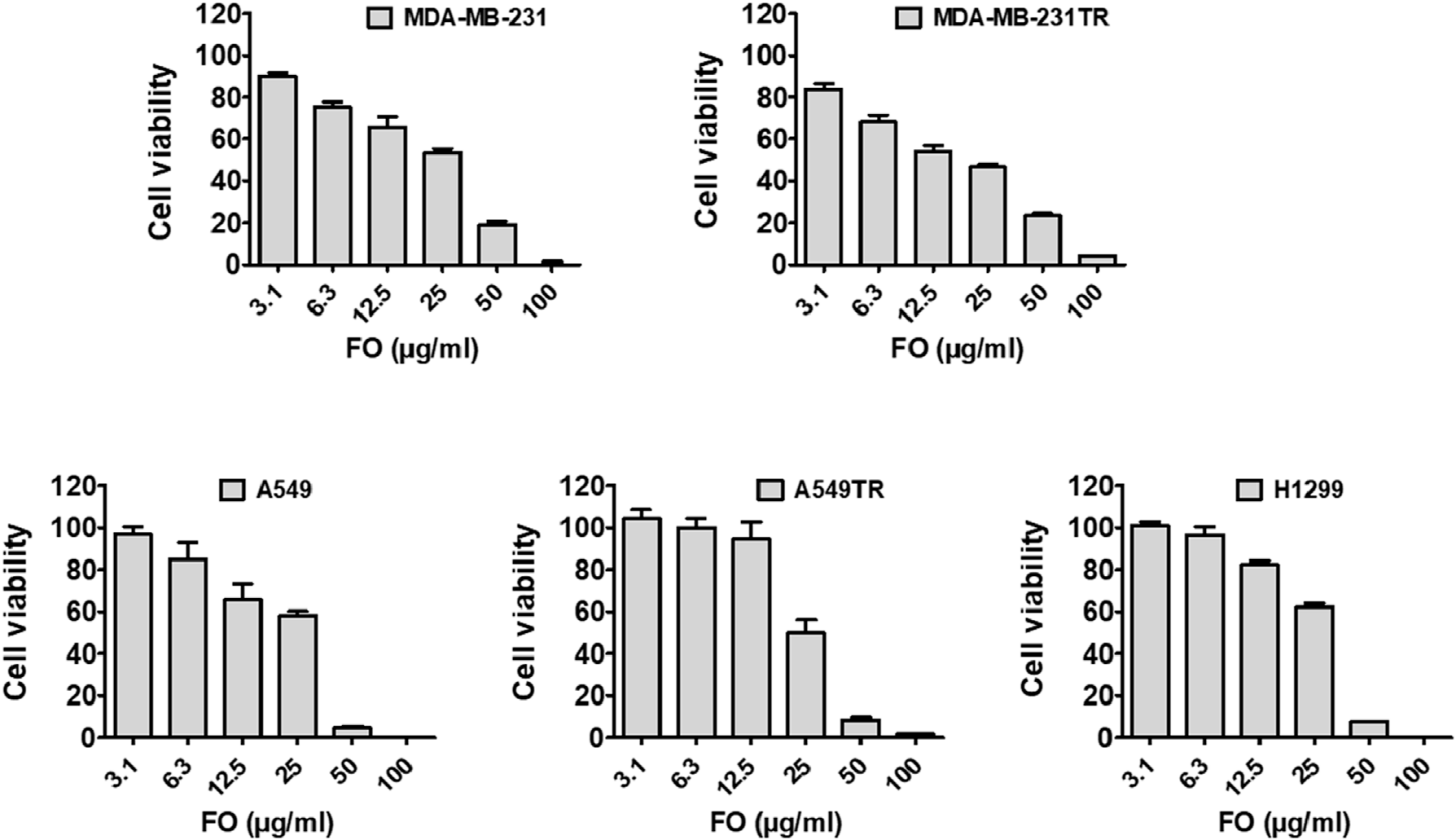
Effect of FO against cancer cell viability. FO inhibits the proliferation of various drug-sensitive and resistant cells. After being treated with 72 h, FO dose-dependently inhibited the drug-sensitive breast cancer MDA-MB-231, lung cancer A549, H1299 cells as well as drug-resistant breast cancer MDA-MB-231-TR, lung cancer A549-TR cells.

The ability of FO to inhibit the growth of breast (MDA-MB-231 and MDA-MB-231 TR) and lung cancer (A549, H1299, and A549 TR) cells was determined by MTT assay. In this assay, mitochondrial reductase enzymes in healthy cells convert the MTT into purple-colored formazan crystals, which is not observed when the cells are dead (Ogunc et al., 2017). FO extract inhibits the growth of both drug-sensitive and resistant breast (MDA-MB-231 and MDA-MB-231 TR) and lung cancer (A549, H1299, and A549 TR) cells in a dose-dependently manner (Figure 5). Interestingly, FO showed a similar response to paclitaxel-resistant breast and lung cancer cells. The IC_50_ values were in the range of 20–30 µM. From the GC-MS analysis, it was observed that FO extract comprised various phytochemicals which exhibit *in vitro* anticancer activity by inducing apoptosis at G_0_/G_1_ phase by oxidative stress and modulating signaling transduction responsible for inhibiting proliferation, angiogenesis, invasion, and, metastasis like NF-κB pathway, c-jun N-terminal kinase (JNK), and mitogen-activated protein kinase (MAPKK) signaling pathways (Chen et al., 2013; Silva et al., 2021).

**FIGURE 6:**
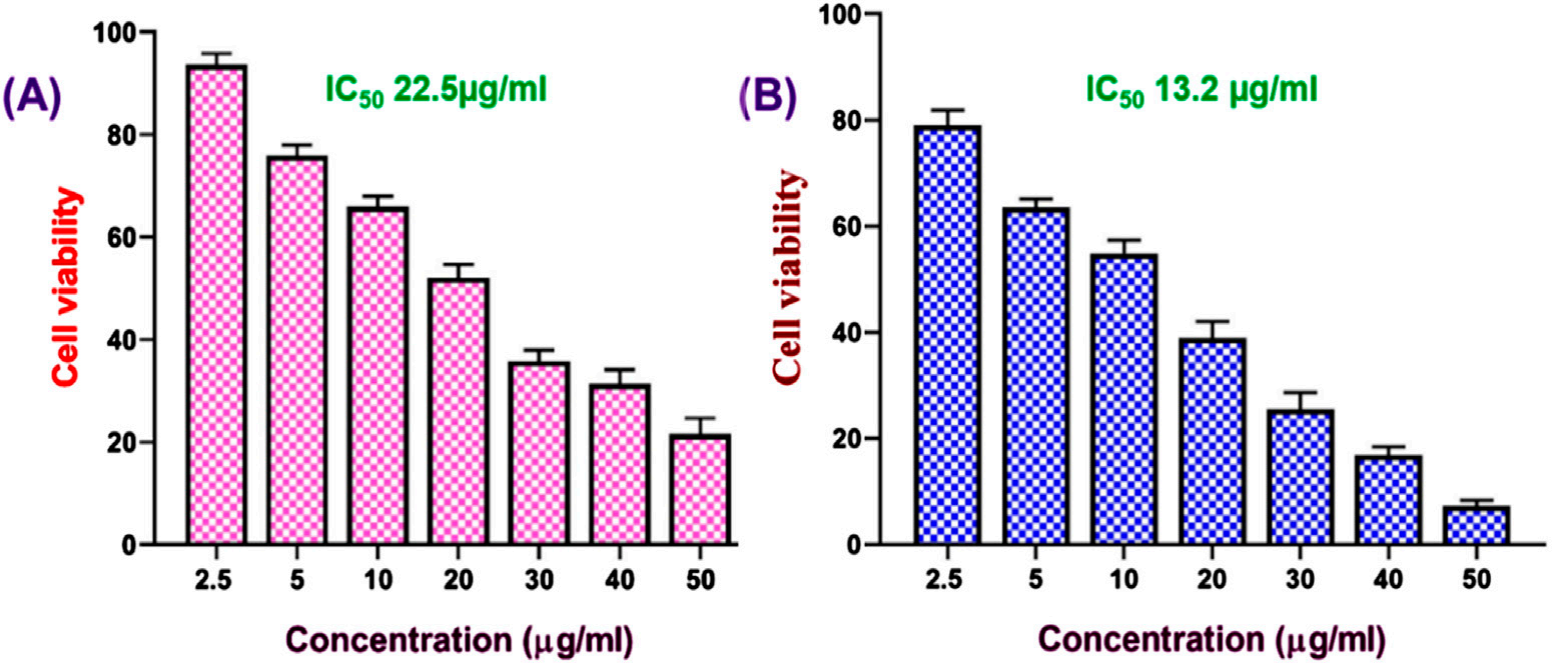
Effect of FO **(A)**, and FO-NEs **(B)** against breast cancer cells. MTT assay revealed that both FO and FO-NEs dose-dependently inhibit the growth of breast cancer cells (MDA-MB-231).

We further assessed the *in vitro* anticancer activity of the FO-NEs using an MTT assay against MDA-MB-231 cell lines for 24 h (Figure 6). The cell line and time were chosen based on the activity of FO alone. From the results, it was observed that FO-NEs (13.2 μg/mL) exhibit higher activity as compared to FO alone (22.5 μg/mL). NEs showed higher activity due its increased internalization, attributed to the amphiphilic nature of surfactants, which aids in mediating an interaction with the lipidic layer of the cell membrane. Also, the NEs increase the membrane fluidity, enhancing the cell membrane’s permeability.

### Wound healing assay

**FIGURE 7:**
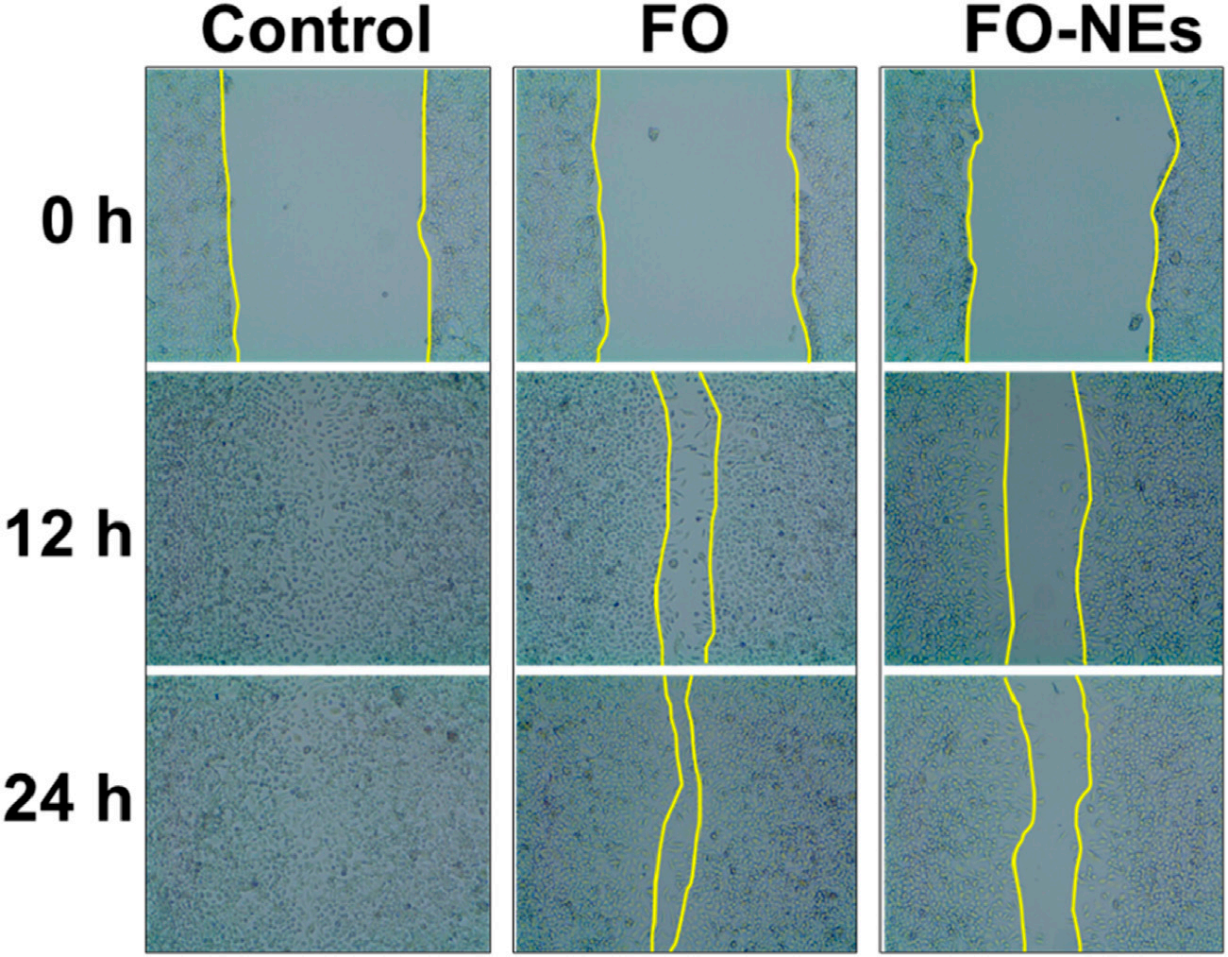
Effect of FO and FO-NEs against breast cancer cell migration. The image depicts the MDA-MB-231 breast cancer cell migration over the course of 24 h following treatment with FO and FO-NEs and comparison with control.

One of the significant challenges in cancer treatment is to control cancer metastasis. Tumor cells, especially solid tumors, can metastasize to different organs (Fares et al., 2020). To evaluate the anti-migratory properties of FO and FO-NEs, they were subjected to wound healing assay or scratch assay, and the results are depicted in Figure 7. It was observed that the cell lines treated with FO showed 20%–30% coverage of the wounded area, demonstrating a significant decrease in migration at 24 h (*p ˂ 0.05). However, the cell lines treated with only media (control group) exhibited 98% coverage of the wounded area, indicating the high migration ability of the cells. But, when the cells were treated with FO-NEs, the wounded area was covered by 60%–70%, indicating a significantly enhanced anti-migratory ability of FO-NEs (*p < 0.05).

### Mitochondrial membrane potential (MMP) assay

Mitochondria release ATP by employing the mitochondrial membrane potential, which is utilized by the cell for extracting energy (McBride et al., 2006). Upon apoptosis, the integrity of the mitochondrial membrane gets disrupted, spilling the mitochondrial content into the cytoplasm of the cell, leading to a disturbance in the mitochondrial membrane potential (Wang and Youle, 2009). Therefore, the condition of the cells could be monitored by analyzing the mitochondrial membrane potential (MMP). JC-1 is a cationic membrane–permeant dye used to assess the status of the mitochondria, as it exhibits potential-dependent accumulation in mitochondria. When the MMP is low, JC-1 dye exists as a monomer and emits green fluorescence.

**FIGURE 8:**
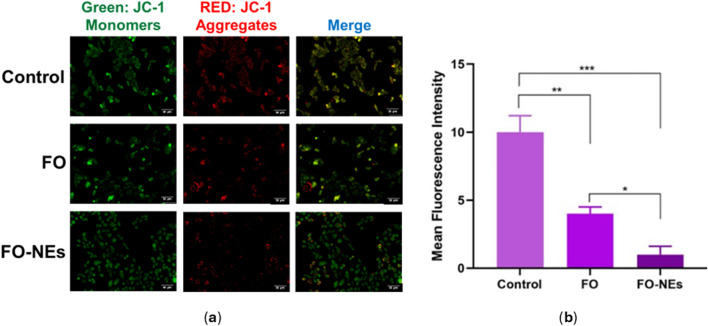
**(A)** Effect of FO, and FO-NEs on mitochondrial membrane potential (MMP) of breast cancer cells (MDA-MB-231), and **(B)** Bar graphs showing the mean fluorescence intensity (MFI) of the control, FO, and FO-NEs as estimated by the red fluorescence (JC-1 aggregates). Scalebar = 50 μm.

On the other hand, as the MMP increases, the JC-1 dye exists as aggregates and emits red fluorescence. Later is also an indication of healthy cells that use the proton pump for generating the ATP. Figure 8 indicates that compared to untreated cells, FO and FO-NEs both lower the MMP of MDA-MB-231 breast cancer cells. FO-NEs significantly reduced the generation of JC-1 aggregates, as compared to free FO, indicating cells treated with FO-NEs underwent apoptosis and exhibited increased *in vitro* anticancer.

### Reactive oxygen species (ROS) production

**FIGURE 9:**
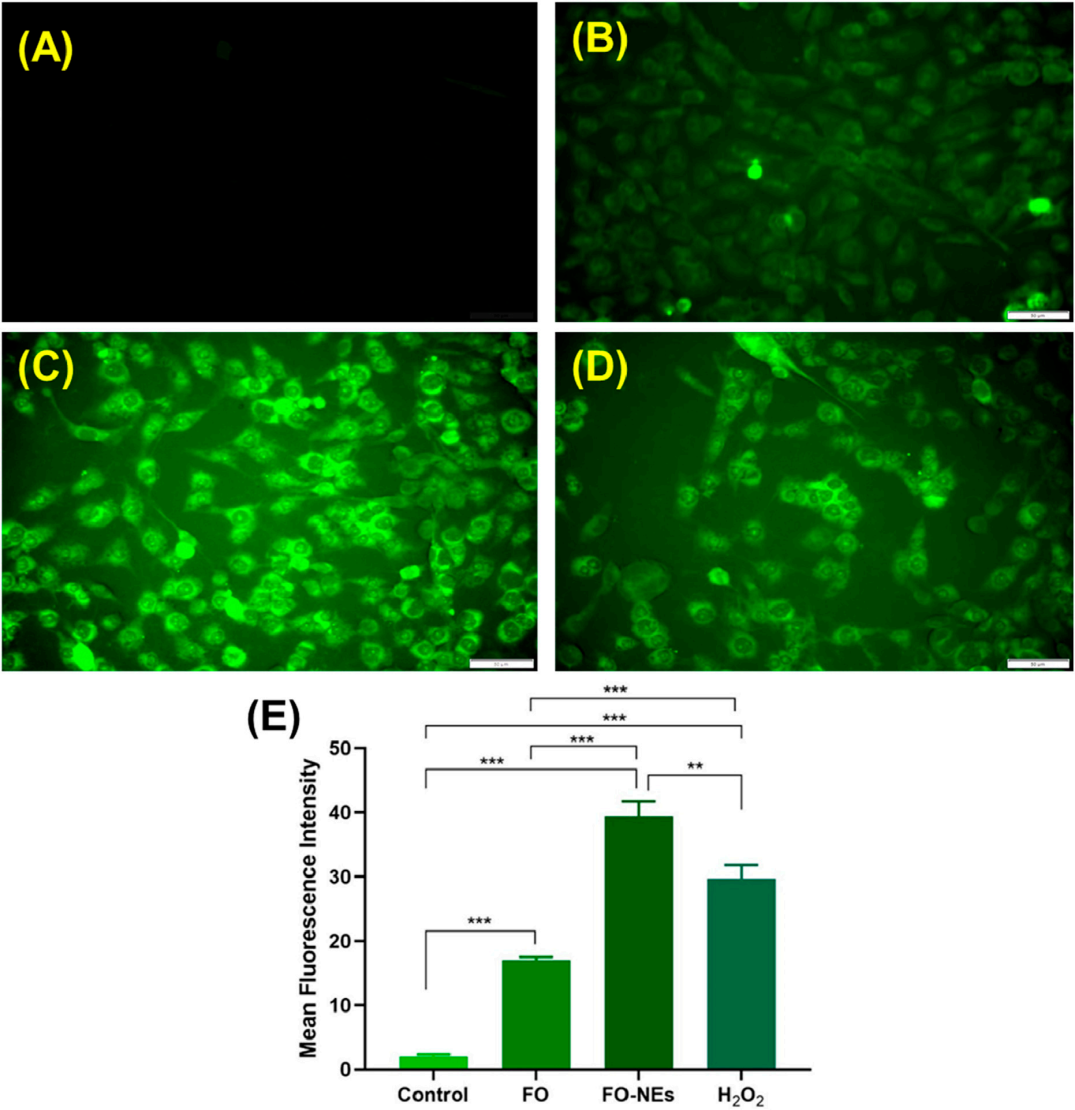
Reactive oxygen species (ROS) assay: **(A)** Healthy cells, **(B)** Cells treated with free FO, **(C)** Cells treated with FO-NEs, **(D)** Cells treated with H_2_O_2_ (positive control). **(E)** Bar graphs showing the mean fluorescence intensity (MFI) of the control, FO, FO-NEs, and H_2_O_2_.

It is well established that the generation of ROS leads to cell damage which eventually causes apoptosis; hence ROS generation plays a pivotal role in cancer studies (Matés and Sánchez-Jiménez, 2000). It was further revealed that the generation of ROS within the cells causes oxidative stress, which alters the redox status and disrupts the biochemical pathways required for survival and growth, destroying the cancer cells (Saneja et al., 2017; Moloney and Cotter, 2018). It was observed that cells treated with FO-NEs generated more ROS than cells treated with free FO, thus revealing the effective *in vitro* anticancer activity of FO-NEs (Figure 9).

## Conclusion

Natural products and nanotechnology are gaining much importance in anticancer therapy, as they show fewer side effects and increased targetability. Using FO as a natural cytotoxic or anticancer agent and delivering it as a nanoemulsion led to exceptional achievement in enhancing the *in vitro* anticancer activity of FO. FO-NEs were prepared and optimized using BBD, where the optimized NEs showed 65.1 ± 4.21 nm particle size (<200 nm), 0.258 ± 0.04 PDI (<0.35), −22 mV zeta potential, and spherical shape, as confirmed by SEM, and AFM. Further, FO showed excellent *in vitro* anticancer activity as revealed through cytotoxicity assay (MTT assay), wound healing assay, mitochondrial membrane potential assay, and reactive oxygen species assay. In conclusion, current research findings exhibited that FO-NEs hold a remarkable anticancer potential that could be employed as a valuable strategy for the management of breast cancer.

## Data Availability

The original contributions presented in the study are included in the article/Supplementary Material, further inquiries can be directed to the corresponding author.
