# Supplementary material for: In vitro anticancer effects of frankincense and its nanoemulsions for enhanced cancer cell targeting
Source: Front Pharmacol. 2025 Feb 6;16:1403780. doi: 10.3389/fphar.2025.1403780 (PMC11839425; doi:10.3389/fphar.2025.1403780)
Supplement: Supplementary file 1 [file DataSheet1.docx]

**Frankincense Nanoemulsions for Improved Cancer Cell Targeting**

Rayya A. Al-Balushi^*,1^**,** Aiswarya Chaudhuri^2^, Raghuram Kandimalla^3^, Ashanul Haque,^4^ Khalaf M. Alenezi,^4^ Mohd. Saeed^5^, Mohammad Changez^6^, Thuraya Al Harthy^1^, Mohammed Al Hinaai^1^, Samra Siddiqui,^5^ Ashish Kumar Agrawal^2^, Farrukh Aqil^3,7^

^1^ Department of Basic and Applied Sciences, College of Applied and Health Sciences, A’Sharqiyah University, P.O. Box 42, Ibra 400, Sultanate of Oman.

^2^ Department of Pharmaceutical Engineering & Technology, Indian Institute of Technology (BHU), Varanasi 221005, India

^3^ Brown Cancer Center, University of Louisville, Louisville, KY 40202, USA

^4^ Department of Chemistry, College of Science, University of Hail, Kingdom of Saudi Arabia.

^5^ Department of Biology, College of Science, University of Hail, Kingdom of Saudi Arabia.

^6^College of Health Sciences, University of Buraimi, Al Buraimi, Oman

^7^Department of Medicine, University of Louisville, Louisville, KY 40202, USA

* Correspondence: [rayya.albalushi@asu.edu.om](mailto:rayya.albalushi@asu.edu.om) (RA)

**Supporting information’s**


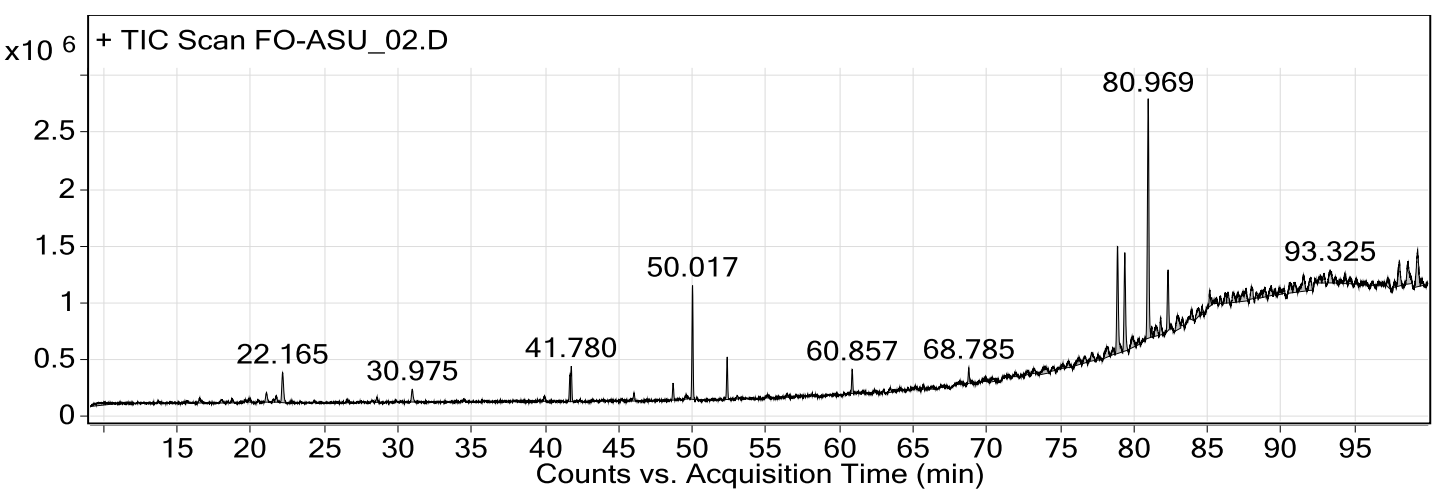


**Figure S1**: Gas chromatography–mass spectrometry (GC-MS) profile of FO extract.

**Figure S2.** FTIR-ATR spectrum of FO extract.


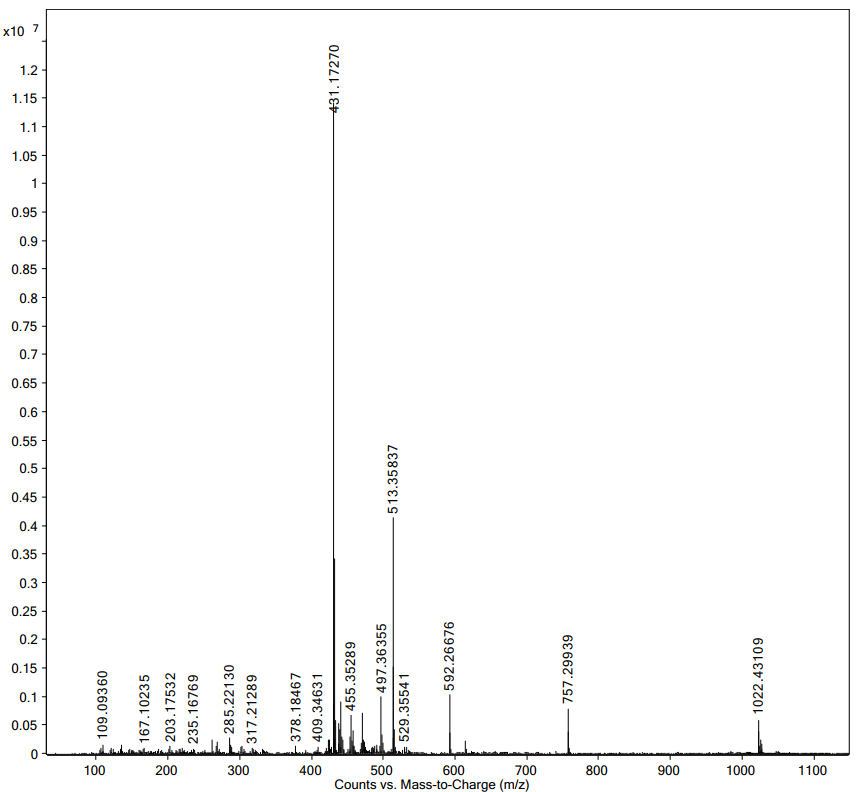


**Figure S3**: LC-MS (positive mode) traces of FO extract.

**Table ST1.** Levels of independent variables and dependent responses applied in BBD

| Independent Variables | Factor Levels | | |
| --- | --- | --- | --- |
|  | -1 | 0 | +1 |
| Concentration of Oil (A) | 5 | 10 | 15 |
| Concentration of Smix (B) | 20 | 30 | 40 |
| Sonication amplitude (C) | 20 | 45 | 70 |
| Dependent responses | Constraints | | |
| Particle size (Y_1_) | < 200 nm | | |
| Polydispersity index (PDI) (Y_2_) | <0.35 | | |

**Table ST2.** Randomized experimental runs in the BBD along with their experimental values.

| **Runs** | **A** | **B** | **C** | **Y_1_** | **Y_2_** |
| --- | --- | --- | --- | --- | --- |
| F1 | 0 | +1 | +1 | 52 | 0.133 |
| F2 | 0 | -1 | -1 | 50 | 0.109 |
| F3 | 0 | 0 | 0 | 72 | 0.271 |
| F4 | 0 | 0 | 0 | 82 | 0.282 |
| F5 | +1 | 0 | -1 | 130 | 0.220 |
| F6 | -1 | +1 | 0 | 91 | 0.267 |
| F7 | 0 | 0 | 0 | 42 | 0.182 |
| F8 | -1 | -1 | 0 | 30 | 0.172 |
| F9 | +1 | -1 | 0 | 75 | 0.154 |
| F10 | +1 | 0 | +1 | 66 | 0.189 |
| F11 | 0 | +1 | -1 | 152 | 0.386 |
| F12 | 0 | 0 | 0 | 50 | 0.236 |
| F13 | 0 | +1 | 0 | 55 | 0.256 |
| F14 | -1 | 0 | +1 | 20 | 0.167 |
| F15 | 0 | -1 | +1 | 42 | 0.221 |
| F16 | -1 | 0 | -1 | 102 | 0.299 |
| F17 | +1 | +1 | 0 | 104 | 0.293 |

A: oil phase concentration (% v/v); B: Smix concentration (%v/v); C: sonication amplitude; Y_1_: droplet size (nm); Y_2_: PDI

$$Y= \beta o+\sum_{i=1}^{k} \beta iXi+\sum_{i=1}^{k} \beta ii{Xi}^{2}+\sum_{1\leq i\leq j}^{k} \beta ijXiXj+e$$

Where, βo, βi, βii, and βij represented the regression coefficients of, constant, linear, quadratic, and interactions terms, respectively, Xi, and Xj represented the independent variables, k represented the number of variables and Y indicated the predicted response. ANOVA (Analysis of Variance) was performed to affirm the fitted mathematical model. Moreover, the accuracy of the model was expressed via regression coefficient (R2), its significance, and lack of fit analysis. Also, three-dimensional response surface graphs were plotted to represent the influence of the interactions between the responses (1).

**References**

1. Fachel FNS, Medeiros-Neves B, Dal Pra M, Schuh RS, Veras KS, Bassani VL, et al. Box-Behnken design optimization of mucoadhesive chitosan-coated nanoemulsions for rosmarinic acid nasal delivery-In vitro studies. Carbohydr Polym. 2018;199:572-82.
